# Interaction between Ammonium Toxicity and Green Tide Development Over Seagrass Meadows: A Laboratory Study

**DOI:** 10.1371/journal.pone.0152971

**Published:** 2016-04-01

**Authors:** Francisco Moreno-Marín, Juan J. Vergara, J. Lucas Pérez-Llorens, Morten F. Pedersen, Fernando G. Brun

**Affiliations:** 1 Department of Biology, Faculty of Marine and Environmental Sciences, University of Cadiz, Campus de Excelencia Internacional del Mar (CEIMAR), 11510 Puerto Real (Cadiz), Spain; 2 Department of Natural Science and Environment, Roskilde University, PO Box 2660, 4000 Roskilde, Denmark; Seagrass Ecosystem Research Group, Swansea University, UNITED KINGDOM

## Abstract

Eutrophication affects seagrasses negatively by increasing light attenuation through stimulation of biomass of fast-growing, bloom-forming algae and because high concentrations of ammonium in the water can be toxic to higher plants. We hypothesized nevertheless, that moderate amounts of nitrophilic macroalgae that coexists with seagrasses under eutrophic conditions, can alleviate the harmful effects of eutrophication on seagrasses by reducing ammonium concentrations in the seawater to non-toxic levels because such algae have a very large capacity to take up inorganic nutrients. We studied therefore how combinations of different ammonium concentrations (0, 25 and 50 μM) and different standing stocks of macroalgae (i.e. 0, 1 and 6 layers of *Ulva* sp.) affected survival, growth and net production of the seagrass *Zostera noltei*. In the absence of *Ulva* sp., increasing ammonium concentrations had a negative influence on the performance of *Z*. *noltei*. The presence of *Ulva* sp. without ammonium supply had a similar, but slightly smaller, negative effect on seagrass fitness due to light attenuation. When ammonium enrichment was combined with presence of *Ulva* sp., *Ulva* sp. ameliorated some of negative effects caused by high ammonium availability although *Ulva* sp. lowered the availability of light. Benthic microalgae, which increased in biomass during the experiment, seemed to play a similar role as *Ulva* sp.–they contributed to remove ammonium from the water, and thus, aided to keep the ammonium concentrations experienced by *Z*. *noltei* at relatively non-toxic levels. Our findings show that moderate amounts of drift macroalgae, eventually combined with increasing stocks of benthic microalgae, may aid seagrasses to alleviate toxic effects of ammonium under eutrophic conditions, which highlights the importance of high functional diversity for ecosystem resistance to anthropogenic disturbance.

## Introduction

Seagrass based ecosystems are among the most productive coastal ecosystem types providing a broad range of ecosystem services such as carbon burial, amelioration of natural hazards and habitat and nursery functions [1,2,3]. These ecosystems are increasingly endangered by anthropogenic pressures; 78% of the human population lives within 50 km of coastline [4] and increasing population density in coastal zones promotes an increase in nutrient loads derived from catchment areas and sewage, which contributes to boost eutrophication processes [5,6]. Eutrophication has a strong negative effect on seagrass systems [2,7,8], which are affected by increased nutrient availability in two major ways. The first and most important is by triggering blooms of fast-growing micro- and macroalgae [9,10]. Such blooms cause increased light attenuation in the water column, which may lead to diminished depth limits [11]. Algal blooms may also result in enhanced inputs of organic matter to the seafloor, leading to sediment anoxia [12] and higher sulphide levels in the sediment pore water, increasing the risk of sulphide intrusion into the plants [13,14,15]. Secondly, eutrophication is typically followed by higher concentrations of dissolved inorganic nutrients in the recipient [16,17], and in the case of ammonium, high concentrations can be potentially harmful (i.e. toxic) to seagrasses [17,18,19].

The negative (toxic) effect of high ammonium concentration has been documented in several studies [19,20,21] where seagrasses exposed to high ammonium availability show slower growth and reduced survival. The toxic effect of ammonium is mainly related to an uncoupling of ATP production from photosynthetic electron transport [22,23], enhanced respiratory demand [24,25], alteration of intracellular pH [23] and decreased uptake of some cations [17,26], all of which may lead to reduced plant performance.

Accumulation of fast-growing micro- and macroalgae and the resulting light attenuation is typically considered more important for seagrass performance than ammonium toxicity under eutrophication, because the concentration needed for ammonium to be toxic is relatively high (typically > 25 μM) and not very common for extended periods of time in nature. Ammonium toxicity is therefore mainly considered relevant in places with extreme inputs of nutrient rich freshwaters and limited water exchange, for example close to point sources in semi-enclosed estuaries and bays.

High concentrations of ammonium may, however, occur in bottom waters due to decomposition of organic matter. Fast decomposition of sediment organic matter combined with anoxic conditions in summer may accelerate the efflux of ammonium from the sediment, resulting in elevated levels of ammonium in near bottom waters where concentrations can reach 50–100 μM [27]. Hence, ammonium concentrations in bottom waters surrounding seagrasses can reach levels at which plants performance is affected. In addition, seagrass meadows are often covered by fast-growing, drift macroalgae under eutrophic conditions [28,29,30,31]. These mats of drift macroalgae reduce light penetration, which may reinforce the toxic effect of ammonium on seagrasses [32,33,34]. A previous study [34] reported a negative synergetic effect of high ammonium concentration and artificially reduced light levels and hypothesized that seagrasses, which are temporarily covered by algal mats in summer, might suffer from two stressors simultaneously since the algal mat attenuates light, while the concentration of ammonium below the mat and around the plants may increase substantially. However, fast-growing, mat-forming macroalgae, such as members of the genera *Ulva*, take up nutrients much faster than seagrasses [35,36,37]. Ammonium uptake by the overgrowing mat-forming algae could thus potentially aid to reduce the average ammonium concentration in the canopy, thereby alleviating the toxic effect of high ammonium concentration on seagrasses. Mat-forming macroalgae may thus have an overall negative effect on the seagrasses they cover (through light attenuation), but may, at the same time, aid seagrasses to sustain prolonged coverage by lowering the exposure to high ammonium concentrations.

We conducted a laboratory experiment where we simulated eutrophic conditions in a shallow water coastal system to investigate both, single and combined effects of high ammonium concentration, and cover by free-floating macroalgae, on seagrass survival and growth. Our aim was to test the hypothesis that the single effect of each stressor on *Zostera noltei* Hornem was negative, but that moderate amounts of *Ulva* sp. could have an ameliorating effect on seagrass performance under high ammonium concentrations.

## Materials and Methods

### Plant, water and sediment collection

Specimens of *Z*. *noltei* were collected at Santibáñez intertidal mudflats (Cádiz Bay Natural Park (36° 28’ N, 6° 15’ W), Spain) with permission granted by "Junta de Andalucía" and "Cádiz Bay Natural Park”. The area is characterized by a mild climate with annual mean water and air temperatures of 19 ± 3 and 17 ± 5.3°C, respectively [38]. Ambient nutrient concentrations in the water column are relatively low, averaging 1.89 ± 1.22 μM ammonium, 0.18 ± 0.14 μM nitrite, 0.68 ± 1.88 μM nitrate and 0.38 ± 0.28 μM phosphate on an annual basis [S1 File]. Experimental plants (*Z*. *noltei*) and algae (*Ulva* sp.–likely *Ulva rigida*) were collected haphazardly from a large area (ca. 20,000 m^2^) and immediately transported to the laboratory. Sediment and water for the experiments was collected from Rio San Pedro, a sandy inlet in the vicinity of the laboratory. Seawater was collected using a pump with a 100 μm filter to remove suspended material.

### Experimental set-up

The experiment was designed to investigate how the combined effect of high ammonium seawater concentrations and the presence of free-floating algae (here *Ulva* sp.) affected survival and growth of *Z*. *noltei*. We used a 2-factorial orthogonal design with 3 levels of ammonium loading and 3 levels of *Ulva* sp. biomass. Each treatment combination was replicated 3 times, rendering a total of 27 aquaria.

The experiment was conducted in a temperature controlled climate room set at 20°C. The aquaria were illuminated by lamps with cool fluorescent tubes (T5 High Output Blau Aquaristic aquarium color extreme fluorescents) in a 16:8 light:dark cycle, resulting in a light intensity of ca. 160 μmol photons · m^2^ · d^-1^. Each aquarium (volume = 20 L) was first filled with ca. 5 L of pre-sieved (through a 0.5 cm mesh) sediment and subsequently filled with ca. 15 L of seawater. Aeration pits were placed in all aquaria to ensure adequate mixing of water and air. Aquaria were left for 24 hours until re-suspended sediment had settled prior to transplantation of the plants.

All *Z*. *noltei* plants were first ‘standardized’ to consist of 1 apical shoot bearing a rhizome with 2–3 intact internodes and associated roots. Plants were individually labeled and then weighted (initial fresh weight; FW_0_) and the initial number of leaves and rhizome internodes per plant were recorded. The plants were subsequently distributed randomly among the 27 aquaria at a density of 15 plants per aquarium, corresponding to a density of ca. 200 shoots m^-2^ or a biomass of ca. 45 g FW m^-2^, which was approximately similar to the plant density and biomass at the sampling site.

**Fig 1:**
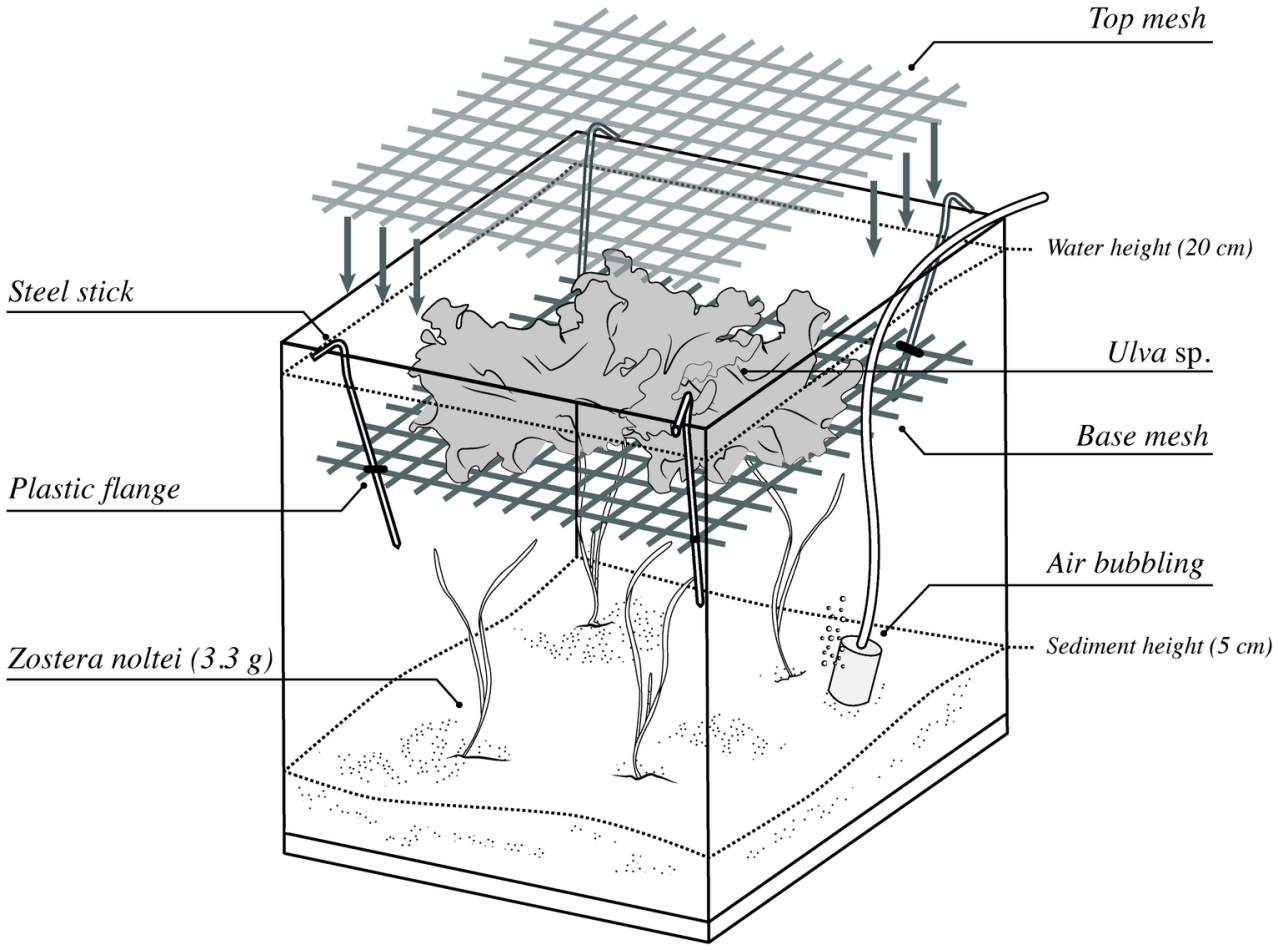
Aquarium set-up.

Layers of *Ulva* sp. (0, 1 or 6 depending on treatment level) were placed between 2 pieces of nylon mesh (mesh size: 1 cm), which was fixed to the walls of each aquarium in the upper part of the water column, allowing the free circulation of seawater around the thalli (Fig 1). The biomass of *Ulva* sp. in treatments with 1 and 6 layers corresponded to a biomass of ca. 38 and 225 g FW aquarium^-1^, or 520 and 3086 g FW m^-2^, respectively, which resemble biomass values of drift algae in eutrophic systems [28,31,39]. The light intensity measured at the sediment surface differed substantially among *Ulva* treatments; the intensity was 28.9 ± 12.2 μmol photons · m^-2^ · s^-1^ (i.e. close to the compensation point of *Z*. *noltei* [40]) with 6 layers of *Ulva* sp., 74.5 ± 21.9 μmol photons ·m^-2^ · s^-1^ with 1 layer of *Ulva* sp. and 158.0 ± 40.3 μmol photons · m^-2^ · s^-1^ in the absence of *Ulva* sp. (saturating light levels). This set up was left for 48 hours to allow plants and algae to acclimate to experimental conditions before initiating the experiment.

Three levels of ammonium loading were assayed; aquaria in the first level (designated as level C) received no additional ammonium, while aquaria in the second treatment level (designated as +N) received 1125 μmol ammonium per week and aquaria in the third treatment level (+NN) received 2250 μmol ammonium per week. Ammonium was added from a stock solution to all aquaria in the +N and +NN treatments as 3 pulses per week. The target concentrations of ammonium in the 3 treatment levels were thus ca. 0, 25 and 50 μM per pulse, respectively. These concentrations were chosen since concentrations above 25 μM are known to cause adverse effects in *Z*. *noltei* [19,20]. Ammonium was added to the water column just beneath the algal layers and close to the aeration pit to ensure quick diffusion of nutrients in the aquaria. Water samples were taken from each aquarium before and 15 minutes after ammonium addition and immediately frozen at -20°C for later analysis of ammonium. Physico-chemical parameters (i.e. temperature, salinity, oxygen saturation and pH) were monitored when ammonium was supplied to the aquaria.

Addition of ammonium was repeated during days 0, 2 and 5 of each week, while water sampling for nutrient analyses and monitoring of physico-chemical parameters was repeated on days 0, 2, 5 and 7 of each week during the experiment (6 weeks in total). The seawater from all aquaria was renewed weekly (on day 7 each week). During water renewal the aquarium walls were cleaned with soft tissues to remove salt and epiphytes from the walls and detached seagrass leaves were removed. Sediment samples were also taken and frozen at -20°C for later analysis of benthic chlorophyll. *Ulva* sp. thalli in the meshes were replaced by new thalli every second week to maintain experimental conditions (i.e. approximately constant biomass and photosynthetic capacity).

All seagrass plants were harvested at the end of the experiment (plants without leaves were considered dead), cleaned and blotted with a soft paper towel, individually weighted (i.e. final fresh weight; FW_F_) and the number of internodes, shoots and leaves per shoot was counted. Survival rate (SR) was estimated for each aquarium from the number of surviving plants. Relative growth rate (RGR; % change in biomass · individual^-1^ · d^-1^) of all surviving *Z*. *noltei* plants was estimated as:

$$RGR=((FW_{F}-FW_{0})/FW_{0})*100*t^{-1}$$

where *FW*_*F*_ and *FW*_*0*_ are the final and initial blotted fresh weight biomass, respectively, and *t* is the incubation time (in days). Net production (NP) of the seagrass assemblage in each aquarium (g FW · aquarium^-1^ · d^-1^) was estimated as the change in total biomass of the seagrass assemblage over time according to:

$$NP=(\sum FW_{FS}-\sum FW_{0})*t^{-1}$$

where *ΣFW*_*0*_ is the total initial fresh weight biomass of all plants in an aquarium and *ΣFW*_*FS*_ is the final total fresh weight biomass of all surviving individuals in the same aquarium and *t* is the incubation time (in days).

### Laboratory analyses

The ammonium concentration in water samples was determined by the salicylate-hypochlorite method [41]. Net removal (uptake) of ammonium from the water in each aquarium was estimated by subtracting the amount of ammonium present in the aquaria at the end of each week from the amount of ammonium added to the water during the preceding week.

Chlorophyll in the sediment was extracted overnight with methanol and measured spectrophotometrically to obtain total chlorophyll concentrations [42].

### Statistical analyses

Physico-chemical variables (water temperature, salinity, pH and oxygen saturation) and the biomass of benthic microalgae were compared across time and treatments (ammonium: 3 levels and *Ulva* sp. biomass; 3 levels, both considered fixed factors) using repeated measures ANOVA.

Changes in final ammonium concentrations from each week and estimated weekly removal of ammonium by the autotrophic assemblage (i.e. seagrass, *Ulva* sp. and benthic macroalgae) across treatments and time could not be tested by repeated measures ANOVA since several treatment combinations had identical replicate values (i.e. 0 μM ammonium or 100% removal, respectively), and therefore, no variation associated to their treatment means. Mean values for each treatment combination and sampling time was instead plotted with ±95% confidence limits for visual inspection.

Two factorial ANOVA was used to test the effects of ammonium loading and *Ulva* sp. biomass on seagrass survival, growth and net production. Tukeys test was used to compare levels of treatment factors when main factors (but not interactions) had a significant effect. In case of significant interactions, Tukeys test was used to compare the levels of each factor within each level of the other factor and *vice versa* using Bonferroni to correct the Type I error level as recommended Underwood (1997) [43] and Meyers et al. 2006 [44]. All data were checked for normality and homoscedasticity using Kolmogorov Smirnoffs test and Levenes test, respectively, and data were ln-transformed when necessary to obtain homogeneity of variances. The level of significance was set at 5% (α = 0.05) in all analyses except those that were Bonferroni corrected.

Pearsons correlation analysis was used to test the possible correlation between ammonium concentration in the water at the end of the week and the biomass of benthic microalgae at the same time. Only data obtained from aquaria without *Ulva* sp. were included in this analysis because presence of *Ulva* sp. would have confounded the outcome of the test.

All statistical analyses were conducted using SPSS v. 22 while graphs were made using SigmaPlot v. 11.

## Results

### Physico-chemical variables

Water temperature averaged 20.9 ± 1.7°C, salinity 38.9 ± 2.9, oxygen saturation 94.7 ± 1.1% and pH 8.3 ± 0.1 across all aquaria and sampling dates. Repeated measures ANOVA did not reveal any significant differences in these variables among treatment combinations nor over time (*p* always >> 0.05).

### Ammonium dynamics in the aquaria

**Fig 2:**
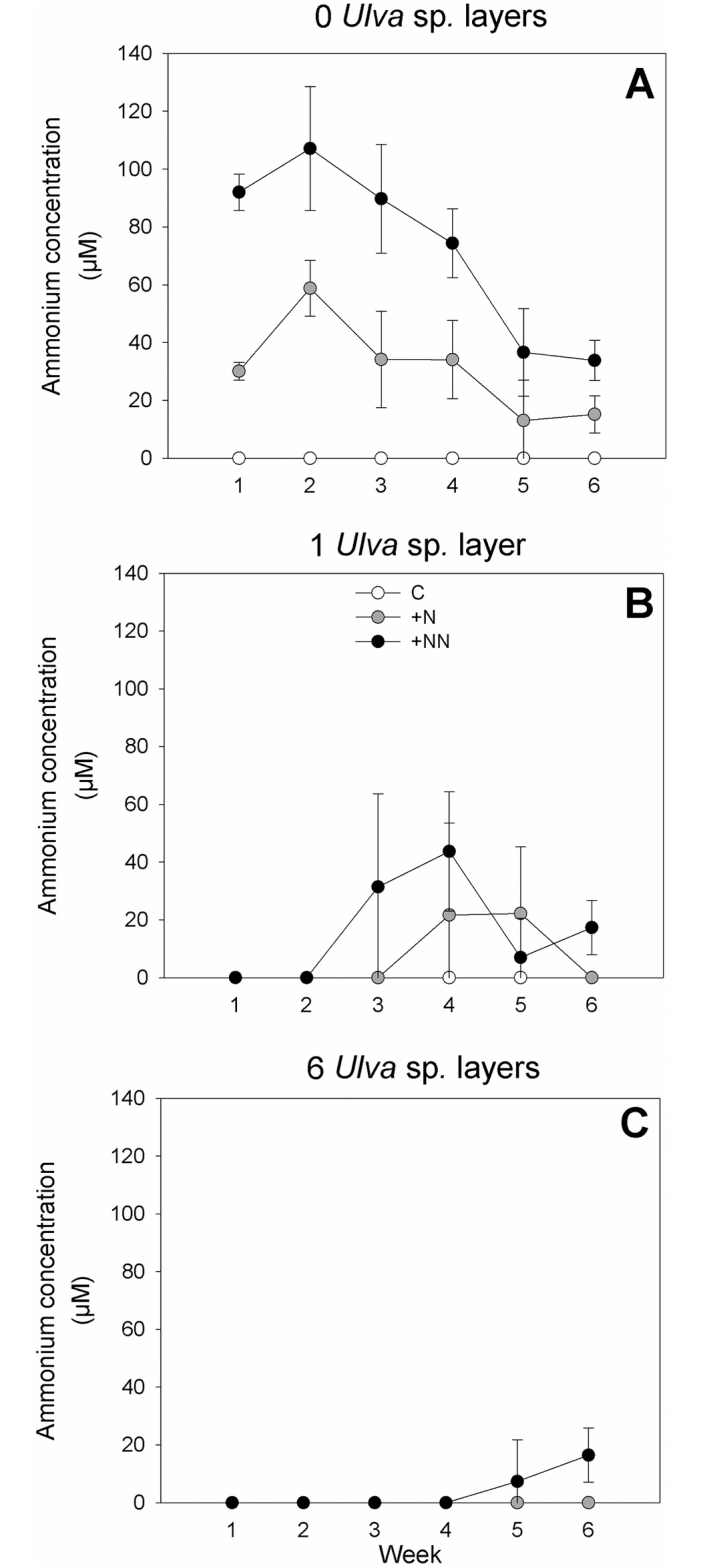
Change in ammonium concentrations at the end of the week over time (μM). A) No *Ulva* sp., B) 1 layer of *Ulva* sp., C) 6 layers of *Ulva* sp. Mean values ± 95% confidence limits (n = 3).

The ammonium concentration in the water at the end of each week (i.e. just before changing the water) differed considerably depending on tretament (Fig 2). Final ammonium concentrations increased generally with increasing ammonium loading, averaging 0 ± 0 μM (mean ±95% CL) in treatments without ammonium addition (across all levels of *Ulva* sp. and sampling time), 12.7 ± 17.4 μM in the +N treatments and 30.9 ± 36.3 in the +NN treatments. Ammonium concentrations at the end of the week tended to decrease with increasing biomass of *Ulva* sp. (across all levels of ammonium and sampling time), averaging 34.4 ± 35.8 μM in treatments without *Ulva* sp., 8.0 ± 13.4 μM in treatments with one layer of *Ulva* sp. and 1.3 ± 4.2 μM with six layers of *Ulva* sp. The effect of ammonium loading tended to diminish with increasing *Ulva* sp. biomass even though the interaction between ammonium and *Ulva* treatments could not be tested formally.

**Fig 3:**
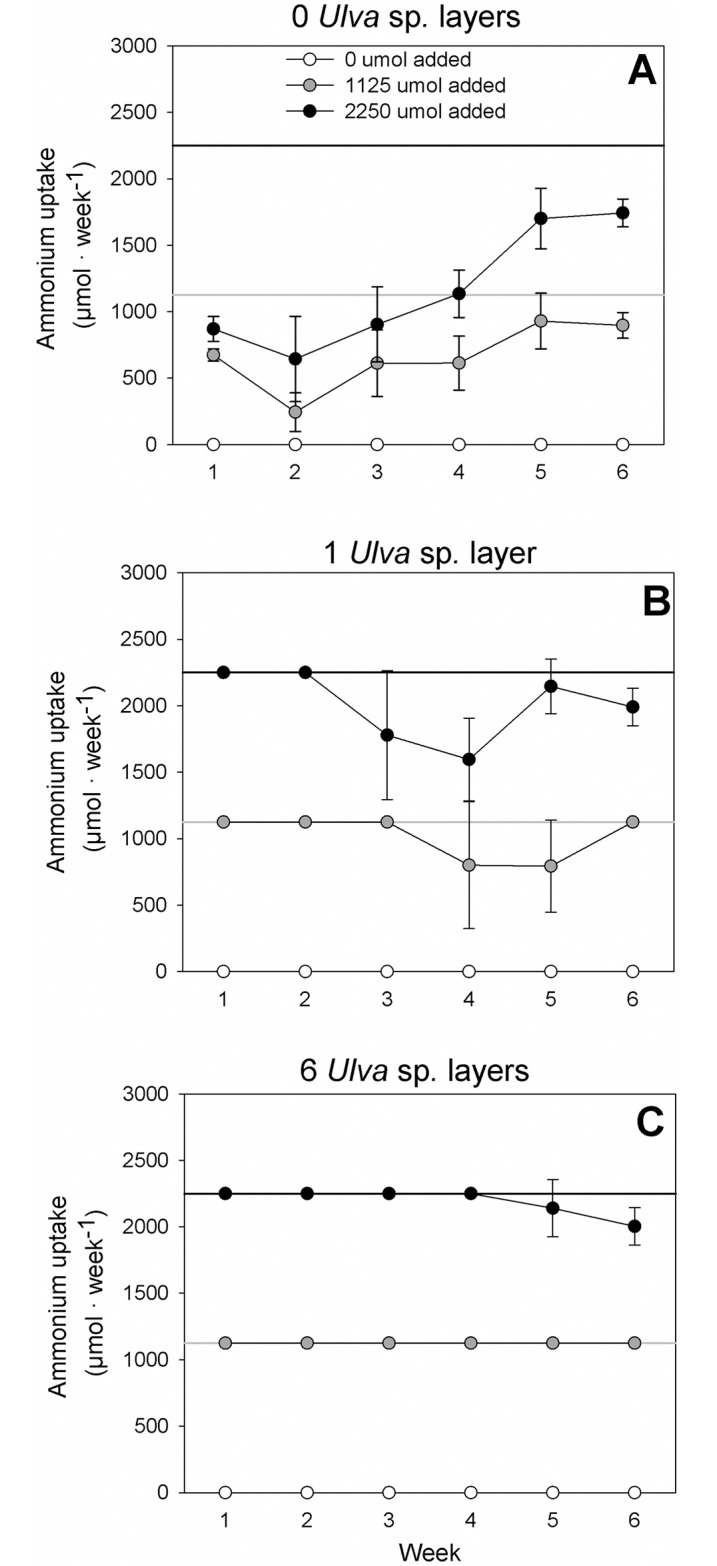
Removal (uptake) of ammonium from the water by autotrophs. A) No *Ulva* sp., B) 1 layer of *Ulva* sp., C) 6 layers of *Ulva* sp. Horizontal lines represent 100% removal of ammonium by autotrophs for the +N treatment (grey) and the +NN treatment (black), respectively. Mean values ± 95% confidence limits (n = 3).

The final concentrations of ammonium in the various treatment combinations reflect the balance between ammonium added (through treatment) and ammonium removed by the autotrophic assemblage (seagrasses, *Ulva* sp. and benthic microalgae) in each aquarium. Estimated ammonium uptake by the autotrophs differed markedly among treatment combinations (Fig 3). Thus, it increased generally with increasing ammonium loading; averaging (across all levels of *Ulva* sp. biomass and sampling time) 0 ± 0 μmol aquarium^-1^ week^-1^ (mean ±95% CL) with no addition of ammonium, 934 ± 261 μmol aquarium^-1^ week^-1^ in the +N treatment and 1786 ± 544 μmol aquarium^-1^ week^-1^ in the +NN treatment. The uptake of ammonium tended to increase with increasing *Ulva* sp. biomass; averaging 609 ± 567 μmol aquarium^-1^ week^-1^ without *Ulva* sp., 1006 ± 858 μmol aquarium^-1^ week^-1^ with one layer of *Ulva* sp. and 1105 ± 922 μmol aquarium^-1^ week^-1^ with 6 layers of *Ulva* sp. From Fig 3 there is no visible indication of a strong interaction effect between ammonium loading and *Ulva* sp. biomass.

### Benthic microalgae

**Fig 4:**
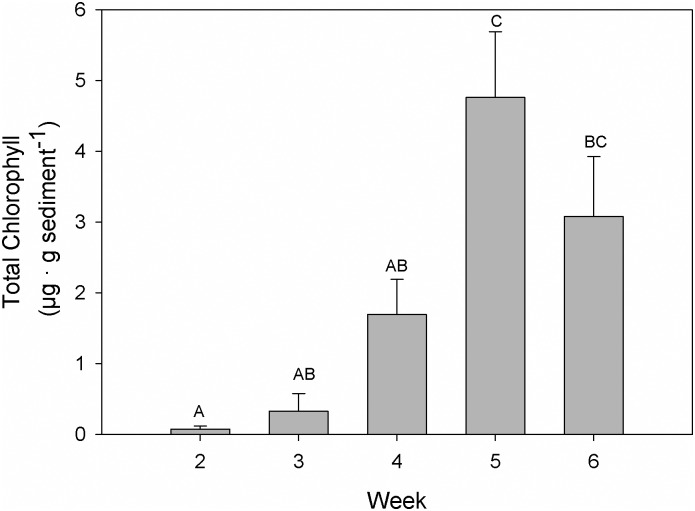
Biomass of benthic microalgae. Each bar represents pooled data from all treatments since there were no significant differences in biomass among treatments. Letters above the bars represent significant differences among weeks. Mean values ± 1 SE (n = 27).

**Fig 5:**
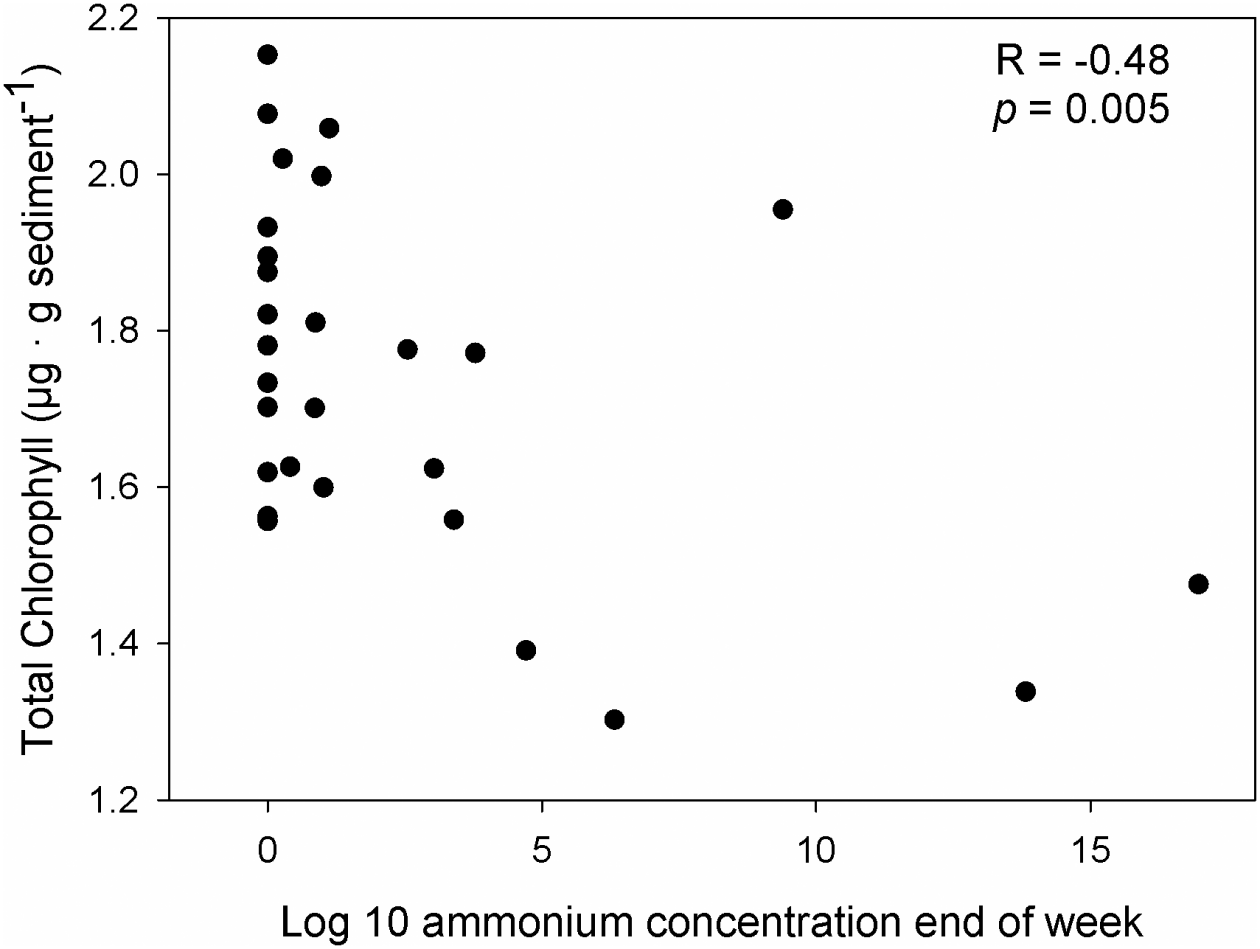
Relationship between ammonium concentration in the water and biomass of benthic microalgae at the end of each week. Only data of treatments without *Ulva* sp. and with ammonium addition were used for this analysis (n = 30).

The average (across all treatment levels) biomass of benthic microalgae (Fig 4) increased significantly from 0.08 ± 0.15 μg Chl g^-1^ sediment (mean ± 1 SE) in week 2 to 4.76 ± 2.36 μg Total Chl g^-1^ sediment in week 5 (RM ANOVA, effect of Time; F = 14.4, *p* < 0.001). The biomass of benthic microalgae was unaffected by ammonium treatment, by *Ulva* treatment and by any of the interactions between ammonium, *Ulva* treatment and time (RM ANOVA, *p* always > 0.066). However, correlation analysis revealed a moderate negative and significant correlation between benthic chlorophyll and ammonium concentration at the end of the week (R = -0.48, *p* = 0.005; Fig 5).

### Plant responses

**Fig 6:**
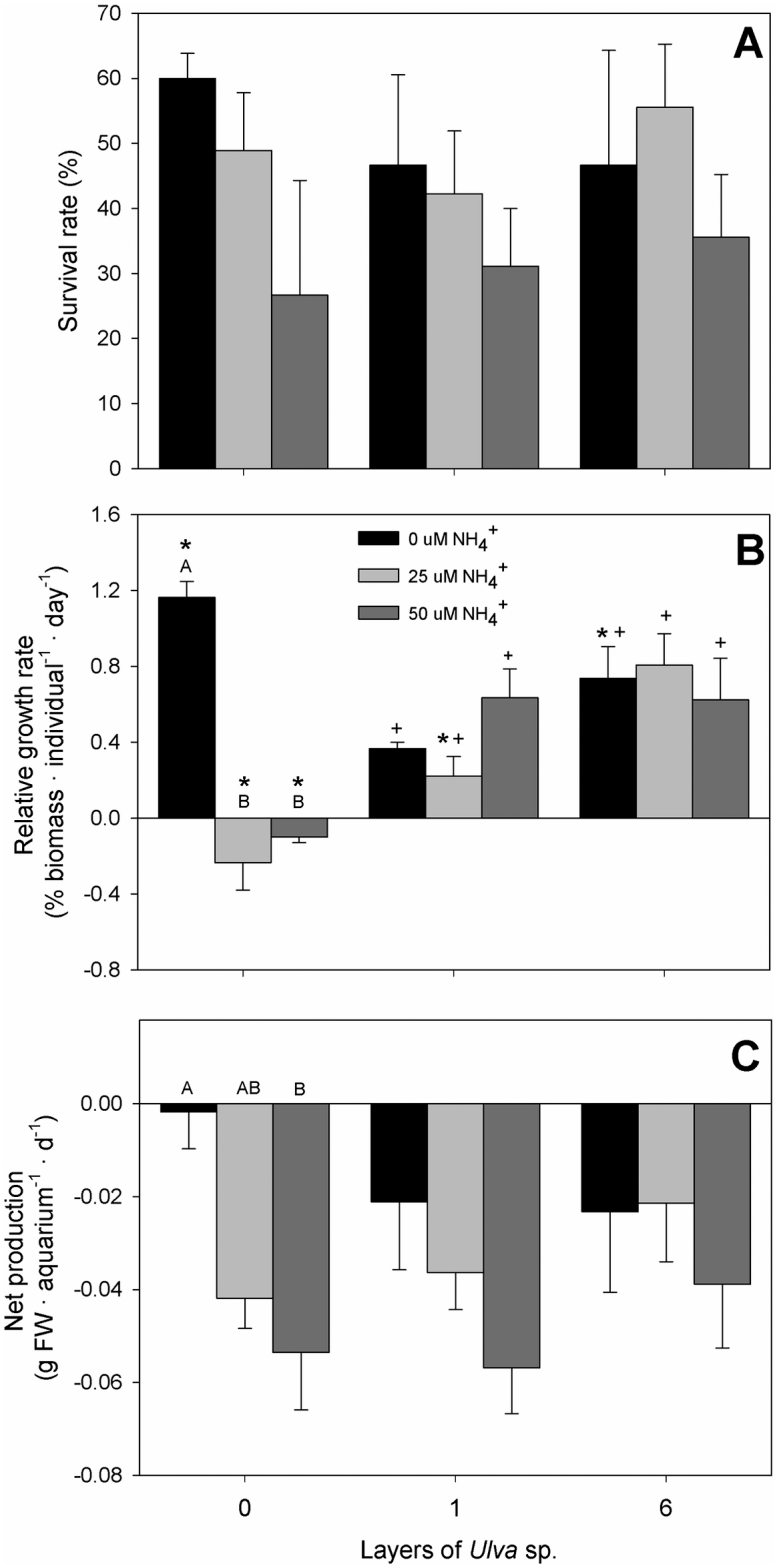
A) Survival rate, B) Relative growth rate and C) Net production of *Z*. *noltei*. Letters over the bars represent significant differences between treatments with the same number of *Ulva* sp. layers; symbols (*, +) represent significant differences between *Ulva* sp. treatments with the same ammonium treatment. Data represent the mean ± SE (n = 3).

*Z*. *noltei* was negatively affected by ammonium addition as indicated by lower survival, slower growth and lower net production in treatments with ammonium enrichment, whereas the presence of *Ulva* sp. tended to alleviate the negative effects of ammonium (Fig 6, Table 1).

**Table 1:** ANOVA results testing the effects of ammonium and *Ulva* treatments on survival rate (SR), relative growth rate (RGR) and net production (NP) of *Zostera noltei*. Bold letters indicate significant differences.

Response	Source	Type III SS	Df	MS	F	*p*
SR:	Ammonium	3.573	2	1.786	2.541	0.107
	*Ulva*	0.384	2	0.192	0.273	0.764
	Ammonium × *Ulva*	2.306	4	0.576	0.820	0.529
	Error	12.656	18	0.703		
RGR:	Ammonium	1.162	2	0.581	10.436	**0.001**
	*Ulva*	0.910	2	0.455	8.174	**0.003**
	Ammonium × *Ulva*	2.648	4	0.662	11.891	**<0.001**
	Error	1.022	17	0.056		
NP:	Ammonium	0.005	2	0.003	6.222	**0.009**
	*Ulva*	0.000	2	0.000	0.561	0.580
	Ammonium × *Ulva*	0.002	4	0.000	0.931	0.468
	Error	0.008	18	0.000		

Survival rate (Fig 6A) ranged from 26.7 ± 30.6% to 60.0 ± 6.7% (mean ± 1 SE) depending on treatment, indicating that there was some mortality in all the aquaria. Survival tended to decrease with increasing ammonium loading across all levels of *Ulva* sp. biomass, albeit this effect was not significant (*p* = 0.107; Table 1). Survival was not affected by *Ulva* sp. biomass nor by the interaction between ammonium loading and *Ulva* treatment (Table 1).

The relative growth rate of *Z*. *noltei* ranged from -0.27% d^-1^ to 1.2% d^-1^ across all treatment combinations and was significantly affected by the interaction between ammonium loading and *Ulva* sp. biomass (Fig 6B, Table 1). Growth rate decreased substantially with increasing ammonium loading in the absence of *Ulva* sp. (C *vs*. +N and C *vs*. +NN; both *p* < 0.001). This negative effect of ammonium loading dissapeared, however, in treatments with 1 or 6 layers of *Ulva* sp., where growth rate did not differ among ammonium treatments (*p* always > 0.138). The effect of *Ulva* sp. biomass on growth was not straightforward; without any addition of ammonium, *Z*. *noltei* grew faster in treatments without *Ulva* sp. than in treatments with 1 layer of *Ulva* sp. (*p* = 0.002), while the opposite was true in treatments with addition of ammonium, i.e. the +N treatment (*p* < 0.001) and the +NN treatment (*p* = 0.006).

Net production was negative in all treatments, and only significantly affected by ammonium loading (Fig 6C, Table 1). Net production decreased with increasing ammonium loading across all levels of *Ulva* sp. biomass (*p* = 0.006). Net production remained unaffected by *Ulva* sp. biomass and it was neither affected by the interaction between ammonium loading and *Ulva* sp. biomass.

We found no significant effects of ammonium loading or *Ulva* sp. biomass, nor any significant effect of the interaction between these main factors on the remaining response variables, i.e. number of new internodes and leaves produced (*p* always >> 0.05, data not shown).

## Discussion

Eutrophication affects seagrasses in several ways. Enhanced light attenuation caused by increasing amounts of phytoplankton, epiphytes and drift macroalgae is considered the most important negative consequence of eutrophication for benthic macrophytes [9,45], but an increasing number of studies have also shown that high ammonium concentrations in the water column can be toxic to seagrasses [18,19,21]. It is however unclear how the combination of high ammonium concentrations and large amounts of drift algae will affect seagrass performance, although recent studies have shown that low light conditions might intensify the negative effect of high ammonium availability [34].

### The effect of high ammonium availability

High ammonium concentrations in the water had a profound negative effect on seagrass performance in our experiment. *Zostera noltei* suffered higher mortality, slower growth and lower net production when cultivated alone (i.e. without *Ulva* sp.) under high ammonium concentrations (i.e. 25 and 50 μM treatments) than when grown without additional addition of ammonium. It agrees with results from other studies on *Z*. *noltei* [17,19,20] and other seagrass species [18,21,34] and confirms that high levels of ammonium in the water can be harmful to *Z*. *noltei*.

Ammonium toxicity appears when plants are exposed to high levels of ammonium in the water for extended periods. In seagrasses, foliar ammonium uptake is rather proportional to the concentration of ammonium in the surrounding water [35,46,47]. Exposure to high concentrations of ammonium will therefore lead to enhanced uptake, assimilation (i.e. production of amino acids) and protein synthesis. Intracellular levels of ammonium are typically held low by assimilation, which continuously removes ammonium from the intracellular space, but ammonium may accumulate within cells when assimilation and protein synthesis slows down as plants become N-replete [48,49] or if assimilation becomes limited by lack of C-skeletons due to reduced photosynthesis and/or depletion of internal carbon stores [17]. Accumulation of intracellular ammonium may alter pH and enzyme kinetics, and thus, adversely affect plant metabolism including photosynthesis [50].

*Zostera noltei* was able to remove most, but not all, of the added ammonium when cultured without *Ulva* sp. (Fig 3A) as indicated by the relatively high ammonium concentrations in the water at the end of each week (Fig 2A). The high ammonium concentrations observed in aquaria with ammonium addition indicate that plants became saturated with nitrogen over the course of the experiment, and that ammonium may have accumulated within the plants. We did not measure internal N-concentrations, nor did we measure internal sugar levels in the plants, but other studies have shown that accumulation of various N fractions (i.e. ammonium, amino acids etc.) in plants and algae appears at time scales of days to few weeks when exposed to high concentrations of ammonium [17, 48], and that decreasing rates of N-assimilation and increasing accumulation of ammonium occurs parallel with decreasing amounts of stored carbohydrates [17]. We feel therefore confident that the observed negative effects of high ammonium concentrations on *Z*. *noltei* were caused by toxic effects due to accumulation of ammonium within the plant cells.

### Presence of *Ulva* and the effect of reduced light conditions

Photosynthesis and growth depends essentially on light and reduced light levels will therefore slow down net photosynthesis and growth. Shading is therefore considered one of the most harmful stressors for seagrasses and other benthic macrophytes. The presence of *Ulva* sp. in the aquaria caused a significant light attenuation and the reduction in light intensity was directly proportional to the number of *Ulva* sp. layers. With 1 layer of *Ulva* sp., light levels at the bottom of the aquaria were reduced to sub-saturating levels and the growth rate of *Z*. *noltei* was reduced by ca. 68% in comparison to the control treatment (i.e. no *Ulva* sp.). Survival and net production tended also to decrease although these rates did not differ significantly from rates in the control treatment without *Ulva* sp.

Light levels were substantially reduced below 6 layers of *Ulva* sp. and corresponded more or less to the compensation point of *Z*. *noltei* [40]. We expected therefore to detect a significant effect on seagrass performance, but the effect was much less evident than with 1 layer of *Ulva* sp. and neither survival, growth nor net production differed significantly from those in the control situation. Although this response seemed puzzling, it could be explained by the fact that *Z*. *noltei* can acquire DOC released from macroalgae and use it as a supplement to DIC obtained by photosynthesis. Previous studies [32,51,52] have shown that growth of *Z*. *noltei* are severely reduced when shaded by 2 layers of *Ulva rigida*, but also, that a larger biomass of macroalgae (i.e. 8 layers of *U*. *rigida)* had an ameliorating effect on seagrasses even though light limitation was stronger. The same authors showed that *Z*. *noltei* was able to acquire DOC released from *U*. *rigida* and that high DOC concentrations in the water stimulated the growth of *Z*. *noltei* significantly under low light conditions.

### The combined effect of *Ulva* sp. and high ammonium levels

The negative effect of high ammonium availability can be boosted under experimentally reduced light intensity using shading screens resulting in a negative synergistic effect of high ammonium and low light [17, 53]. Combining the two main factors (i.e. high ammonium level and shading caused by overlying *Ulva* sp.) resulted however in a quite different response in this experiment. The growth rate of *Z*. *noltei* increased significantly at high ammonium availability when moderate amounts of *Ulva* sp. were present, and a similar trend was observed for survival and net production albeit these differences were not statistically significant. This positive effect of *Ulva* sp. can be explained by the interaction between main factors; addition of ammonium to cultures without *Ulva* sp. resulted in saturated uptake of ammonium in *Z*. *noltei* that experienced relatively high concentrations of ammonium over the course of the experiment. In contrast, when cultured together with *Z*. *noltei*, *Ulva* sp. contributed to remove ammonium from the water, and thus, prevented ammonium concentrations to increase to critical, toxic levels within *Z*. *noltei*.

*Ulva* sp. and other fast-growing ephemeral macroalgae take up ammonium much faster than seagrasses per unit time and biomass [35,36,47]. The potential removal of ammonium by *Z*. *noltei* and *Ulva* sp. in treatments with both species can be estimated from the observed biomass and published uptake kinetic values (i.e. V_max_ and K_m_) for *Ulva lactuca* [36] and leaves of *Zostera marina* [47]. Assuming that plants and algae were exposed to either 25 or 50 μM ammonium, *Z*. *noltei* would be able to remove ca. 234 or 270 μmol ammonium aquarium^-1^ d^-1^ (assuming a biomass equivalent to the initial biomass per aquarium), which is less than added to each aquarium in the two treatments (375 and 750 μmol ammonium every second day, respectively), explaining why ammonium concentrations could remain high in treatments without *Ulva* sp. Seagrass biomass declined over the course of the experiment in all treatments except those with no ammonium addition, so these estimates are conservative and likely overestimating the actual removal of ammonium by *Z*. *noltei* during the last 3–4 weeks of the experiment. The potential removal of ammonium by *Ulva* sp. was 40–250 fold higher than that for *Z*. *noltei*, being 10.6 and 11.7 mmol d^-1^ (in the 25 and 50 μM treatments, respectively) with one layer of *Ulva* sp. and 62.7 and 69.4 mmol d^-1^ with six layers of *Ulva* sp. *Ulva* sp. had thus a great potential to remove ammonium from the water and it is evident that 1 and especially 6 layers of *Ulva* sp. must have been able to keep concentrations of ammonium low to the benefit of *Z*. *noltei* that performed better in the presence of *Ulva* sp. even though light conditions were deteriorated. A study on freshwater macrophytes in a Chinese hyper-eutrophic lake supports our findings. The authors of this study [54] found that the concentration of ammonium in the water column decreased substantially during an algal bloom, while at the same time, the performance of rooted macrophytes growing in the system was improved when compared to the performance of plants before the algal bloom.

The efficient removal of ammonium from the water in treatments with *Ulva* sp. can be explained by the large uptake capacity of this macroalgal species. Without *Ulva* sp. and partly 1 layer of *Ulva* sp., final ammonium concentrations at the end of each week were relatively high and especially so during the initial 1–3 weeks of the experiment, after which they decreased progressively over the course of the experiment. This would suggest that the capacity of *Z*. *noltei* to remove ammonium increased with time. The capacity to acquire ammonium from the water should, however, decrease as plants become more N-replete [49]. Also, the absolute removal of ammonium through uptake should partly depend on seagrass biomass, which decreased significantly in all treatments except those without ammonium addition. The increased capacity to remove ammonium from the water can therefore not be explained by an increased capacity to acquire ammonium by *Z*. *noltei*, nor by an increasing biomass of *Ulva* sp., since the biomass of *Ulva* sp. was kept constant by continuously replacing old thalli by new ones with approximately the same initial biomass. Instead, it seems that the increasing uptake capacity of the autotrophic assemblages can be explained by the increasing biomass of benthic microalgae. The amount of benthic microalgae increased over time and across all treatment combinations (Fig 4). A significant negative correlation between benthic chlorophyll and ammonium concentration in the water column at the end of the week was found, suggesting that part of the ammonium was being depleted by benthic microalgae. The biomass of these algae might have been substantially smaller than that of *Z*. *noltei* and *Ulva* sp., but microalgae grow much faster and, thus, have a much higher need for nitrogen per unit biomass and time [55]. The large potential of benthic microalgae to remove ammonium from the overlying water was shown in a previous study [56] where it was demonstrated that benthic microalgae could remove up to 80% of the ammonium in the water column across a range of concentrations similar to those used in our experiment. Our results therefore suggest that at least 2 (benthic microalgae and *Z*. *noltei*) or 3 (*Ulva* sp., benthic microalgae and *Z*. *noltei*) different types of organisms were involved in the removal of ammonium from the water column and also that further studies are required to investigate the larger capacity to remove ammonium from the water column by more diverse assemblages.

In summary, eutrophication affects seagrasses in various ways: by increasing light attenuation caused by proliferating fast-growing algae (i.e. phytoplankton, epiphytes and drift macroalgae), and through ammonium toxicity when concentrations become high enough. Seagrass meadows in eutrophic, sheltered coastal areas are often covered by fast-growing drift macroalgae in summer [28]. Such algal mats can be thick and they may persist and cover the seagrasses for weeks. Under such conditions, light attenuation through the mat may be complete, which is certainty harmful to the underlying seagrasses. Algae in the lower part of the mat may also suffer severe light limitation, which may stop growth and nutrient uptake, and, thus, create a barrier for the ammonium that diffuses from the sediment. Ammonium may thus accumulate to high concentrations in the water surrounding the canopy. Seagrasses covered by thick layers of drift macroalgae may therefore suffer from substantial light limitation and potential ammonium toxicity at the same time. In contrast, when algal mats are thinner and/or are more dynamic [31], algae in the lower part of the mat will still receive light (albeit at lower levels than those the upper part of the mat). All algae in the mat will be actively growing [57], and they will take up nutrients, which may aid to remove part of the ammonium diffusing from the sediment thereby contributing to keep the ammonium concentrations relatively low around the seagrass plants. We hypothesize that moderate layers of drift macroalgae may aid seagrasses to sustain shorter periods of algal cover through removal of excess ammonium although such seagrasses will be negatively affected by the algal cover through light limitation.

## Supporting Information

S1 File Personal communication letter from Gloria Peralta confirming that the data I provide in the manuscript are correct. (DOCX)
