# Supplementary material for: Interaction between Ammonium Toxicity and Green Tide Development Over Seagrass Meadows: A Laboratory Study
Source: PLoS One. 2016 Apr 1;11(4):e0152971. doi: 10.1371/journal.pone.0152971 (PMC4817992; doi:10.1371/journal.pone.0152971)
Supplement: S1 File — (DOCX) [file pone.0152971.s001.docx]

Departamento de Biología


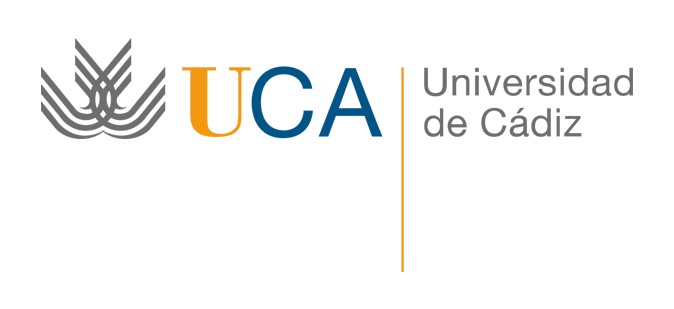

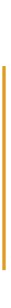


Área de Ecología

Gloria Peralta González

Facultad de Ciencias del Mar y Ambientales

11510 Puerto Real (Cádiz)

tel. 956 016 428 - fax. 956 016 019

e-mail: [gloria.peralta@uca.es](mailto:peralta@uca.es)


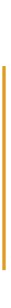


To whom it may concern:

Puerto Real, June 26th, 2015

I, Gloria Peralta González, declare that the data related to nutrient concentrations in the sampling area cited in this manuscript were supplied by me, and therefore, I agree that Francisco Moreno use them as personal communication.


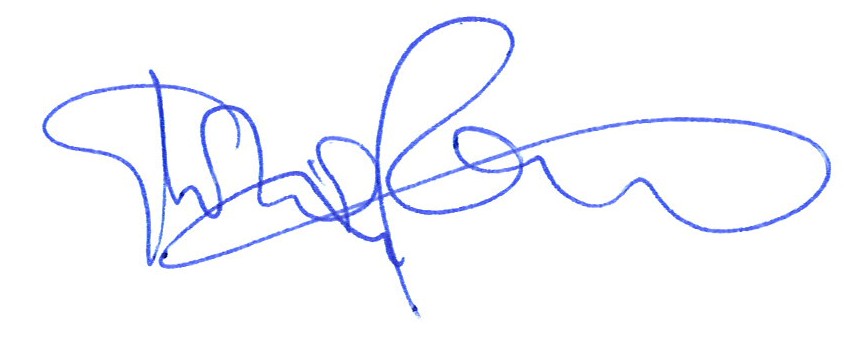


Gloria Peralta

June 26, 2015
